# A single test approach for accurate and sensitive detection and taxonomic characterization of Trypanosomes by comprehensive analysis of internal transcribed spacer 1 amplicons

**DOI:** 10.1371/journal.pntd.0006842

**Published:** 2019-02-25

**Authors:** Alex Kiarie Gaithuma, Junya Yamagishi, Axel Martinelli, Kyoko Hayashida, Naoko Kawai, Megasari Marsela, Chihiro Sugimoto

**Affiliations:** 1 Division of Collaboration and Education, Research Center for Zoonosis Control, Hokkaido University, Sapporo, Japan; 2 GI-CORE, Research Center for Zoonosis Control, Hokkaido University, Sapporo, Japan; 3 Biological and Environmental Sciences and Engineering (BESE) Division, King Abdullah University of Science and Technology (KAUST), Thuwal, Saudi Arabia; Hunter College, CUNY, UNITED STATES

## Abstract

To improve our knowledge on the epidemiological status of African trypanosomiasis, better tools are required to monitor Trypanosome genotypes circulating in both mammalian hosts and tsetse fly vectors. This is important in determining the diversity of Trypanosomes and understanding how environmental factors and control efforts affect Trypanosome evolution. We present a single test approach for molecular detection of different Trypanosome species and subspecies using newly designed primers to amplify the Internal Transcribed Spacer 1 region of ribosomal RNA genes, coupled to Illumina sequencing of the amplicons. The protocol is based on Illumina’s widely used 16s bacterial metagenomic analysis procedure that makes use of multiplex PCR and dual indexing. Results from analysis of wild tsetse flies collected from Zambia and Zimbabwe show that conventional methods for Trypanosome species detection based on band size comparisons on gels is not always able to accurately distinguish between *T*. *vivax* and *T*. *godfreyi*. Additionally, this approach shows increased sensitivity in the detection of Trypanosomes at species level with the exception of the *Trypanozoon* subgenus. We identified subspecies of *T*. *congolense*, *T*. *simiae*, *T*. *vivax*, and *T*. *godfreyi* without the need for additional tests. Results show *T*. *congolense* Kilifi subspecies is more closely related to *T*. *simiae* than to other *T*. *congolense* subspecies. This agrees with previous studies using satellite DNA and 18s RNA analysis. While current classification does not list any subspecies for *T*. *godfreyi*, we observed two distinct clusters for these species. Interestingly, sequences matching *T*. *congolense* Tsavo (now classified as *T*. *simiae* Tsavo) clusters distinctly from other *T*. *simiae* Tsavo sequences suggesting the *Nannomonas* group is more divergent than currently thought thus the need for better classification criteria. This method presents a simple but comprehensive way of identification of Trypanosome species and subspecies-specific using one PCR assay for molecular epidemiology of trypanosomes.

## Author summary

Tsetse flies are central actors in the transmission of Trypanosomes to vertebrate hosts. Therefore, detection of Trypanosomes in the tsetse flies is important for understanding the epidemiology of African trypanosomiasis as a component of new control or surveillance strategies. We have developed a method that combines multiplex PCR and next-generation sequencing for the detection of different Trypanosome species and subspecies. Similar to the widely used bacterial metagenomic analysis protocol, this method uses a modular, two-step PCR process followed by sequencing of all amplicons in a single run, making sequencing of amplicons more efficient and cost-effective when dealing with large sample sizes. As part of this approach, we designed novel Internal Transcribed Spacer 1 primers optimized for short read sequencing and have slightly better sensitivity than conventional primers. Taxonomic identification of amplicons is based on BLAST searches against the constantly updated NCBI’s *nt* database. Our approach is more accurate than traditional gel-based analyses which are prone to misidentification of species. It is also able to discriminate between subspecies of *T*. *congolense*, *T*. *simiae*, *T*. *vivax*, and *T*. *godfreyi* species. This method has the potential to provide new insights into the epidemiology of different Trypanosome genotypes and the discovery of new ones.

## Introduction

Human African trypanosomiasis (HAT) or sleeping sickness is classified as a neglected tropical disease by WHO, that is endemic in sub-Sahara Africa. HAT affects impoverished rural areas of sub-Saharan Africa, where it coexists with animal trypanosomiasis constituting a major health and economic burden [1]. The disease is caused by protozoan parasites of the genus *Trypanosoma*, it is transmitted by the bite of blood-sucking tsetse flies (Diptera, genus *Glossina*). The human disease is caused by *Trypanosoma brucei rhodesiense* and *Trypanosoma brucei gambiense*, causing an acute and chronic disease in humans respectively [2]. *T*.*b*. *rhodesiense* is found in East Africa and transmitted by *Glossina morsitans*, while *T*.*b gambiense* is distributed in West Africa and is mainly transmitted by *Glossina pallidipes* [3–5]. Uganda is the only country that both forms of the disease occur with the potential for overlapping infections [6]. According to WHO, the incidence of sleeping sickness has fallen over the years, from 10,388 cases reported in 2008 to 2,804 cases reported in 2015 [7]. However, WHO estimates the number of actual cases to be below 20,000 [8]. This decrease is attributed to improved case detection and treatment and vector management [9]. Despite this decreased incidence, it is estimated that up to 70 million people distributed over 1.5 million km^2^ remain at risk of contracting the disease [10]. Besides, African animal trypanosomiasis (AAT) is one of the biggest constraints to livestock production and a threat to food security in sub-Saharan Africa. The parasites *T*. *congolense* (Savannah) and *T*. *vivax* are considered the most important animal Trypanosomes due to their predominant distribution in sub-Saharan Africa and their economic impact [11]. They cause pathogenic infections in cattle (*Nagana*) and also infect sheep, goats, pigs, horses, and dogs, while *T*. *brucei brucei* (and *T*. *brucei rhodesiense*) is pathogenic to camels, horses, and dogs, but causes mild or no clinical disease cattle, sheep, goats and pigs [12–14]. *T*. *simiae* causes a fatal disease in pigs and mild disease in sheep and goats. *T*. *godfreyi* shows a chronic, occasionally fatal disease in pigs experimentally [15,16]. *T*. *evansi* was originally found to infect camels but it is present in dromedaries, horses, and other equines as well as in a wide range of animals causing *Surra* disease, while *T*. *equiperdum* causes dourine in equines [17]. Three species (*T*, *evansi*, *T*. *vivax*, and *T*. *equiperdum*) are independent of the tsetse fly vector and thus distributed outside Africa [18,19]. Their transmission is either mechanically, for *T*. *evansi and T*. *vivax*, or sexually for, *T*. *equiperdum*. *T*. *vivax* can be transmitted cyclically by *Glossina spp*. and mechanically and therefore can found in both tsetse-infested and tsetse-free areas [20]. Given that Trypanosome parasites are maintained in wild and domestic animals as reservoirs, this complicates control measures.

Morphological methods have limited ability to distinguish between Trypanosome species due to the existence of trypanosomes sharing developmental sites, and mixed and immature infections. Thus, molecular methods are used for species identification. Identification of Trypanosome species and subspecies is important to interrogate aspects such as what contribution different species/subspecies make to livestock disease and, are species/subspecies differences responsible for assumed “strain” differences in drug response among others. The ribosomal RNA sequence region harboring internal transcribed spacer sequences have been used to identify Trypanosome species in hosts and vectors. Epidemiological and screening studies rely on polymerase chain reaction (PCR) to amplify the internal transcribed spacer 1 (ITS1) region of ribosomal genes to analyze Trypanosome species diversity [16–19]. This locus located between the 18s and 5.8s ribosomal subunit genes which are about 100–200 copies [21] and is widely used to identify Trypanosome species based on amplicon size in [22] a gel. However, ITS1 PCR coupled with viewing products on agarose gels fails to distinguish some species/genotypes such as *T*. *simiae* and *T*. *simiae Tsavo*. Another limitation with ITS1 PCR is the sensitivity of detection, showing bias in detection of some Trypanosome species over others [23,24]. Some are prone to non-specific amplification particularly in bovine blood samples [25]. To address some of the problems that ITS PCR method poses, fluorescent fragment length barcoding (FFLB) method has been developed for Trypanosome species detection [26]. FFLB is based on length variation in regions of the 18s and 28s ribosomal RNA gene region. Fluorescently tagged primers, designed in conserved regions of the 18s and 28s ribosomal RNA genes, are used to amplify fragments with inter-species size variation, and sizes are determined accurately using an automated DNA sequencer. FFLB has been shown to be more sensitive in the identification of Trypanosome species and subspecies and has the capacity to detect new species through identification of unique barcodes [27,28]. However, the method requires the use of four different PCR reactions per sample. A major problem with identification of Trypanosome species with the use of ribosomal RNA genes is that they cannot be used to distinguish between *Trypanozoon* species (*T*. *brucei brucei*, *T*. *brucei rhodesiense*, *T*. *brucei gambiense*, *T*. *evansi*, and *T*. *equiperdum*) [22,26,29,30]. Currently, Trypanozoon subspecies are identified by specific PCR [31–34] and microsatellites markers [32,35,36].

When dealing with a large number of samples either for tsetse fly or animal infection prevalence studies, undertaking multiple PCRs for each sample is an expensive and a laborious undertaking. Most often PCR amplicons are sequenced to confirm species identification usually through capillary sequencing. Recently, next-generation sequencing (NGS) has been established as a well-established method for profiling bacterial and fungal, communities. Among the many advantages, NGS provides a higher sensitivity to detect low-frequency variants, the lower limit of detection of DNA, higher throughput with sample multiplexing and comprehensive coverage among others. With the exception of *Plasmodium* in mosquitoes, relatively few studies have applied this technology in the diagnostics of protozoal infections [37,38]. It is therefore suited in the analysis of the genetic diversity of Trypanosome genotypes which is a composite aspect of understanding anthropogenic disturbance that may change repertoires of trypanosomes infecting human and livestock [39].

## Materials and methods

### Tsetse fly collection

**Fig 1:**
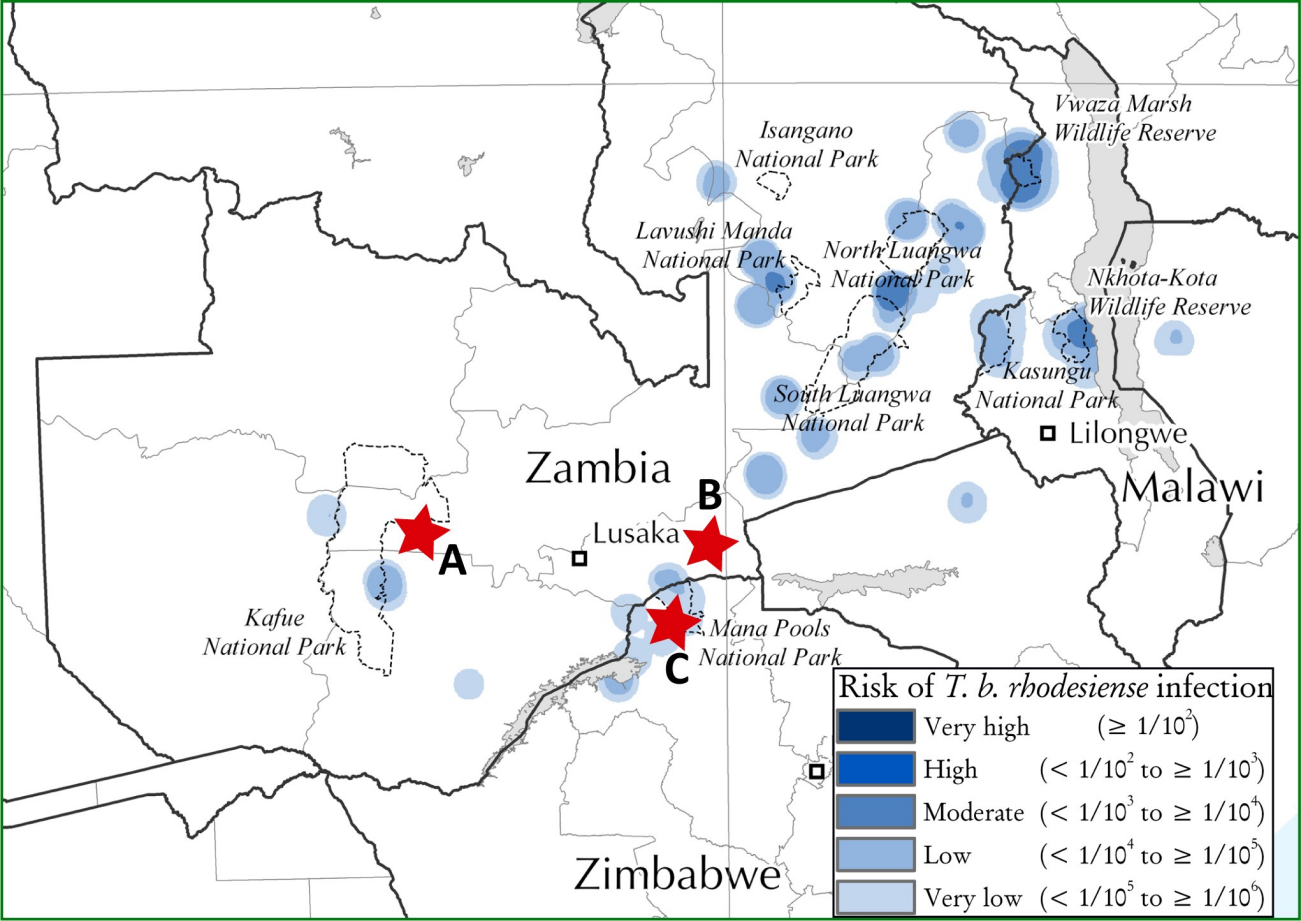
Map of Zambia and Zimbabwe showing areas of tsetse fly collection. The map was sourced from Simarro P, *et al*, 2012 [40] and modified in Adobe Illustrator CC 2019 v23.0.1. Areas where tsetse flies were caught are marked in red stars: (A) Kafue in Zambia, (B) Rufunsa in Zambia and (C) Hurungwe in Zimbabwe.

For this study, we analyzed tsetse fly samples from three different groups collected at three different locations (Fig 1) at different times. The first group was used for the initial analysis and to validate our method and consisted of 188 tsetse flies collected from the area around Hurungwe Game reserve in Zimbabwe between March and April 2014. The second group was included in our final analysis to expand Trypanosome species spectrum and diversity and consisted of 200 tsetse flies from Rufunsa area (Zambia) near Lower Zambezi National park (surrounding farms and villages) collected in November and December 2013). For these samples, information on tsetse fly species and sex was not available. The third group comprised of 85 flies caught in Zambia; on the border between Kafue National park and public settlement area, collected in June 2017. For this group, flies were sorted according to sex and their species identity determined morphologically. Flies from all three groups had been collected using either custom-made mobile traps attached on a slow-moving vehicle (Kafue and Rufunsa groups) or Epsilon traps (Hurungwe group). Individual flies were preserved in separate tubes containing silica gel ready for crushing and DNA extraction. All flies analyzed in this study were caught on public land.

### Extraction of DNA

DNA extraction from all tsetse fly samples analyzed in this study was done by following a protocol adopted for extraction of DNA from crushed tsetse fly samples. Briefly, dried flies in tubes containing stainless beads were transferred to a smashing machine and crushed at 3,000 rpm for 45 sec. DNA from crushed flies was isolated using the DNA Isolation kit for mammalian blood (Roche USA) as per the manufacturer’s protocol with the slight modification suggested for extraction of DNA from Buffy coat, where Red blood cell lysis step is bypassed. This allows lysis of all cells in the solution at once including trypanosomes using the white cell lysis buffer. The DNA sample was stored at -80°C until analysis.

### Primer design

The following sequences were retrieved from NCBI, *Trypanosoma brucei* (JX910378, JX910373, JN673391, FJ712717, AF306777, AF306774, AF306771 and AB742530), *Trypanosoma vivax* (JN673394, KC196703 and TVU22316), *Trypanosoma congolense* (JN673389, TCU22319, TCU22318, TCU22317 and TCU22315), *Trypanosoma simiae* (JN673387 and TSU22320), *Trypanosoma godfreyi* (JN673385) *Trypanosoma evansi* (D89527), *Trypanosoma otospermophili* (AB175625), and *Trypanosoma grosi* (AB175624). They were aligned in Geneious 9.1.5 software (Biomatters Ltd, Auckland, New Zealand) using MAFFT multiple aligner with default settings and ITS1 region identified by comparing annotations and terminal regions of 18s and 1.5s ribosomal RNA regions. Pairs of primers flanking the ITS1 region were picked manually based on the consensus of bases in the alignment flanking the ITS1 region. Manual editing was done on the final primer pair that was chosen, to improve the range of Trypanosome species and subspecies. We used Primer-BLAST (https://www.ncbi.nlm.nih.gov/tools/primer-blast) to confirm that the primers would amplify the target species, check the species range and the melting temperature. The final pair comprised our new primers named Amplification of ITS (AITS) forward (AITSF) and reverse (AITSR).

### Experimental and *in silico* validation of primers

#### *In silico* testing

The expected amplicon sizes for the newly designed AITSF and AITSR primers were compared with amplicons (*in silico*) from other three widely used primers; CF/BR primers [24] and ITS1/ITS2 primers [41]. For *in silico* testing, we used Simulate_PCR [42]; a computer-based PCR analysis algorithm using the NCBI *nt* database to deduce the scope of Trypanosome species and subspecies detection and the expected lengths of amplicons. Simulate_PCR uses BLAST to search amplicons from a specified database wherein we used a local *nt* database downloaded on 3^rd^ December 2017 from NCBI: ftp://ftp.ncbi.nlm.nih.gov/blast/db/. Simulate_PCR was run using the command; *simulate_PCR–db <path/to/database> -primers <path/to/primers*.*fasta>–minlen 100 –maxlen 750 -mm 1 –num_threads 8 –max_target_seq 10000 –genes 1 –extract_amp 1*

#### Sensitivity testing

We also tested the sensitivity of AITSF/AITSR primer set against the CF/BR primer set by PCR to determine their sensitivity in amplifying the ITS1 region of different Trypanosome species. The sequences; *T*. *brucei* (AF306774), *T*. *simiae* (JN673387), *T*. *vivax* (KM391828), *T*. *congolense* (U22317) and *T*. *godfreyi* (JN673384) were downloaded from NCBI, the18s to 5.8s ribosomal RNA region was obtained from each sequence, synthesized and each insert cloned into a pGEMT-easy vector. We chose Primer-BLAST subject sequences common to both primer pairs. Of these, we picked the longer sequences that had a 18s region and complete regions of ITS1 and 1.5s ribosomal RNA. One sequence was picked at random to represent each of the five Trypanosome species from those that passed. For each of the pGEMT-easy vector stock solutions, we calculated the plasmid number per μL (equivalent to ITS1 copies since each plasmid has one copy). Working solutions containing 10^7^ plasmids were prepared then diluted serially to obtain a final dilution with 1 copy of ITS1 insert (1 plasmid). These dilutions were used as templates for PCR reaction (1 μL per reaction) using either AITSF/AITSR or CF/BR primer sets. PCR was done in 10 μL primary reactions containing 0.5 μL of 10 μM each of the forward and reverse primers, 10 μL of 2X Ampdirect Plus buffer, 0.16 μL of 5 U/μL Taq polymerase (Kapa Biosystems, Boston, USA), 0.4 μL DMSO, and 1 μL extracted DNA as a template. The temperature and cycling profile included incubation at 95°C for 10 min, followed by 37 cycles as follows: 95°C for 30 sec, annealing at 60°C for 1 min for AITSF/AITSR primers and 58°C for 1 min for CF/BR primers, 72°C for 2 min, and final extension at 72°C for 10 min. Results were analyzed on 1.5% Agarose gel.

#### Paired-end library preparation

A two-step PCR protocol for the library preparation was applied in the multiplex PCR analysis. We used the newly designed AITSF/AITSR primer set ligated to Illumina adapter sequences (Table 1).

**Table 1:** Primers used in this study.

Description	Primer name	Primer sequence (5'-3')
From Ref [24]	ITS1 CF	CCGGAAGTTCACCGATATTG
	ITS1 BR	TTGCTGCGTTCTTCAACGAA
New ITS1 forward primer	AITSF	CGGAAGTTCACCGATATTGC
New ITS1 reverse primer	AITSR	AGGAAGCCAAGTCATCCATC
Adapter sequence for the forward primer	Illumina adapter forward	ACACTCTTTCCCTACACGACGCTCTTCCGATCT**NN**[AITSF]^a^
Adapter sequence for the reverse primer	Illumina adapter reverse	GTGACTGGAGTTCAGACGTGTGCTCTTCCGATCT**NN**[AITSR] ^a^

^a^ [] indicate where the adapter is attached to the respective primer

ITS1 PCR was done in duplicate for Rufunsa samples to validate Trypanosome detection results. We also included positive template controls comprising. *T*. *b gambiense*, *T*. *b rhodesiense*, and *T*. *congolense* DNA. An artificial Trypanosome DNA mixture was included to mimic a mixed infection control. It comprised artificially mixed *T*.*b*. *gambiense and T*. *congolense* DNA mixed in equal proportions. The controls were processed the same as samples from PCR to sequencing. The first PCR reaction used AITSF/AITSR primers which were ordered in adapter ligated forms where Illumina adapter sequences were added to the 5’ end of each primer (Table 1). Sequencing libraries were prepared according to the Illumina MiSeq system instructions [43].

The first PCR was done in 20 μL primary reactions containing 0.5 μL of 10 μM each of the AITSF and AITSR primers, 10 μL of 2X Ampdirect Plus buffer, 0.16 μL of 5 U/μL Taq polymerase (Kapa Biosystems, Boston, USA), 0.4 μL DMSO, and 1 μL extracted DNA as template. The temperature and cycling profile included incubation at 95°C for 10 min, followed by 37 cycles as follows: 95°C for 30 sec, annealing at 60°C for 1 min, 72°C for 2 min, final extension at 72°C for 10 min.

The second PCR was done in 10 μL reactions containing 1 μL of 10 μM Illumina dual-index primer mix (i5 and i7 primers), 1.2 μL of 25 mM MgCl_2_, 0.4 μL of 10 mM each of the dNTPs, 0.1 μL of 5 U/μL Taq polymerase, 4 μL 5X buffer, and 2 μL of 1/60 diluted primary PCR product as template. The temperature and cycling profile included incubation at 95°C for 3 min, followed by 11 cycles as follows: 95°C for 30 sec, 61°C for 1 min, 72°C for 2 min and a final extension at 72°C for 10 min. A negative template control was included in each set of PCR reactions. To enable the sequencing of all amplicons in this study in one run, we used different sets of dual index primers for each sample in the second PCR reactions.

#### Library sequencing

The barcoded second PCR products were analyzed in 1.5% agarose gel. Equal volumes of each sample were pooled into one library. The library pool was purified using the Wizard SV Gel and PCR Clean-Up System (Promega, Madison, WI, USA) by cutting out bands of interest to separate them from primer dimers and post PCR reagents. Quantification of each of the library was done using a Qubit dsDNA HS assay kit and a Qubit fluorometer (ThermoFisher Scientific, Waltham, MA, USA). The concentration of the library was then adjusted to a final concentration of 4 nM using nuclease-free water and applied to the MiSeq platform (Illumina, San Diego, CA, USA). Sequencing was performed using a MiSeq Reagent Kit for 300 base pairs, paired-end (Illumina, San Diego, CA, USA) and a 20% PhiX DNA spike-in control added to improve the data quality of low diversity samples, such as single PCR amplicons. All controls were also included in the sequencing library.

Raw read data obtained from this study is available at Sequence read archive (SRA) database under the SRA accession number SRP159480 (https://trace.ncbi.nlm.nih.gov/Traces/study/?acc=SRP159480).

#### Bioinformatics

**Fig 2:**
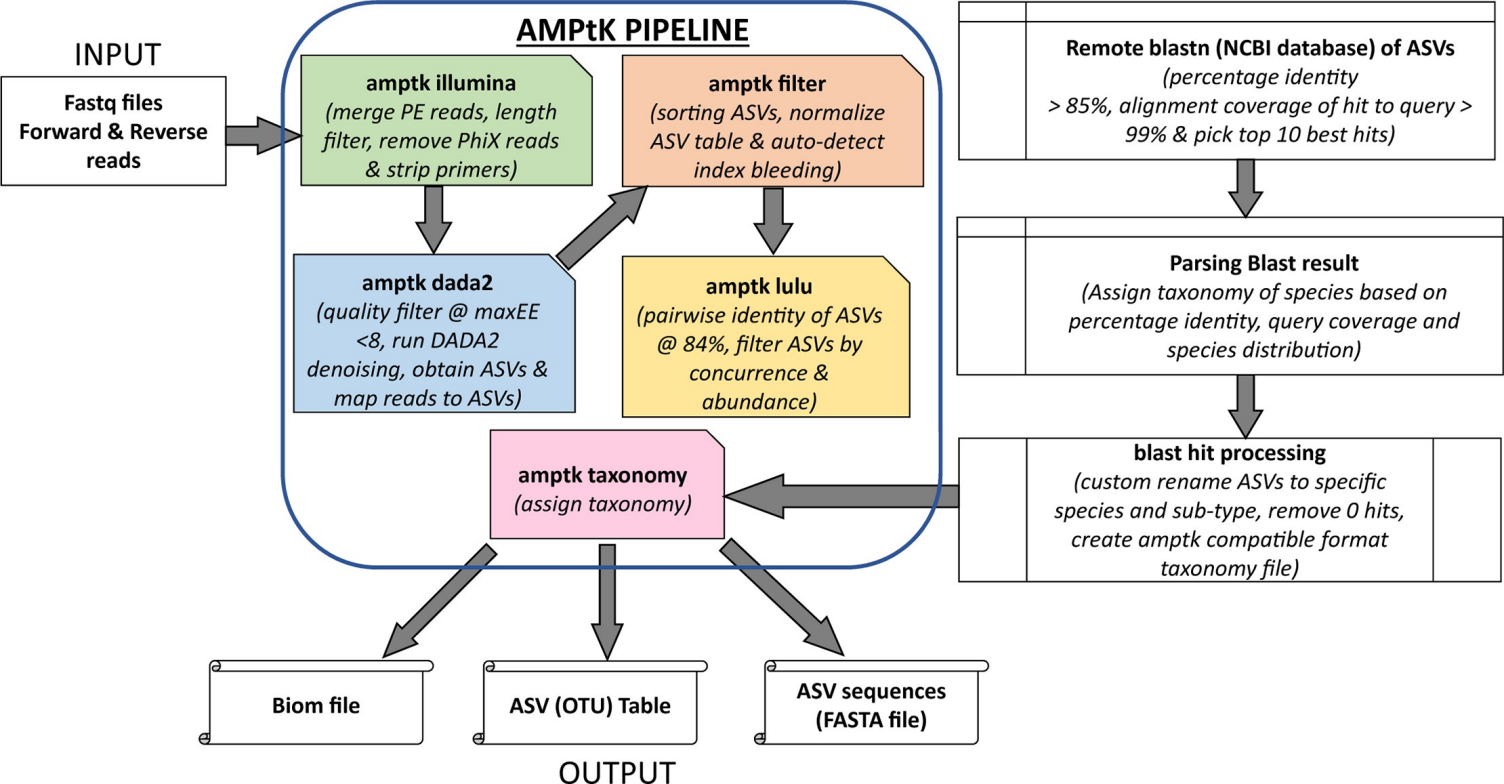
Workflow for read analysis using AMPtk pipeline.

The analysis followed a workflow (Fig 2) comprising the Amplicon Tool Kit (AMPtk) pipeline coupled with taxonomic identification by BLAST. All commands for analysis were run as a custom script (S1 Text). Briefly, reads were processed using the AMPtk pipeline by; 1) Trimming primers, removal of sequences less than 100 b.p, and merging pair-end reads. Merging parameters were customized by editing the AMPtk file amptklib.py with the USEARCH options; *fastq_pctid* set to 80, (minimum %id of alignment), *minhsp* set to 8, and *fastq_maxdiffs* set 10 to limit the number of mismatches in the alignment to 10. 2) Clustering; the denoising algorithm (Divisive Amplicon Denoising Algorithm) DADA2 was called within AMPtk pipeline using the *amptk dada2* command. This algorithm provides a clustering independent method that attempts to “correct” or “denoise” each sequence to a corrected sequence using statistical modeling of sequencing errors. AMPtk implements a modified DADA2 algorithm that produces both the standard “inferred sequences” referred to as amplicon sequence variants (ASVs) output and also clusters the ASVs into biologically relevant operational taxonomic units (OTUs) using the UCLUST algorithm. 3) Downstream processing of ASVs where ASV table filtering was done to correct for index-bleed where a small percentage of reads bleed into other samples. This was done by the *amptk filter* command using 0.005, the default index-bleed percentage. 4) An additional post-clustering ASV table filtering step was done using the *amptk lulu* command. LULU is an algorithm for removing erroneous molecular ASVs from community data derived by high-throughput sequencing of amplified marker genes [44]. LULU identifies errors by combining sequence similarity and co-occurrence patterns yielding reliable biodiversity estimates. 5) Taxonomy was assigned to the final ASV table. ASV taxonomic identification (in this study) was done by BLAST (v2.6.0) [45] remotely using custom options specified as shown in Fig 2. The BLAST output file was parsed and edited to match the taxonomy header formatting specified in the AMPtk manual and subsequently used for generating a taxonomy labeled ASV table.

To check the accuracy of the ASVs generated by the Amptk pipeline, we simulated FASTQ files generated *in silico* from downloaded sequences used in a previous study [15]. This was done by running *ArtificialFastqGenerator* [46], to generate paired-end FASTQ files with 1000 reads per sequence. Real quality scores and simulation of sequencing errors was achieved by using a pair of FASTQ files from sequencing output of the samples. Amptk pipeline was then run on the generated reads. The resultant ASVs were allocated taxonomic identity at species level by BLAST and then compared to the species identity of parent sequences. All the software used in data analysis are free under open access licenses. All ASVs generated in this study are deposited in GenBank submission: SUB4757113 with accession numbers MK131764—MK132190.

#### Phylogenetic and statistical analysis

A phylogenetic tree was created from the alignment generated from ASVs obtained after analysis. Alignments were made with MAFFT [47] using the *mafft-xinsi* option (allowing for prediction of RNA secondary structure and build a multi-structural alignment) with 1,000 maximum iterations, leaving gappy regions and using ‘kimura 1’ option for score matrix. Maximum likelihood phylogenetic trees were built with RAxML 8.0.26 using the 'GTRCATI' model and default parameters with 10,000 bootstraps. The tree was visualized and annotated using iTOL (version 4) [48]. Statistical analysis and graphing of data were carried out in GraphPad Prism version 6.01 for Windows, GraphPad Software, San Diego California USA, www.graphpad.com.

## Results

### Improved primers

*In silico* evaluation of the primers showed that our newly designed primer pair (AITSF/AITSR) had a broad range similar to previously developed ITS1/ITS2 primer set [41] while the range of the CF/BR primer set, previously developed to detect pathogenic Trypanosomes [24] was confined to the pathogenic (S1 Table). We evaluated the sensitivity of newly designed AITSF/AITSR primers to amplify ITS1 region of different Trypanosome species in comparison to commonly used ITS1 primers; CF/BR primers. PCR was performed on pGEMT-easy plasmid DNA containing ITS1 inserts from different Trypanosome species at different dilutions. Our evaluation was based on the visual sight of bands in a gel (the conventional method of analysis). Our results showed that AITSF/AITSR primers were slightly more sensitive in the detection of *T*. *brucei*, *T*. *simiae* and *T*. *congolense* (S1 Fig). AITSF/AITSR primers could detect 10^3^ *T*. *brucei*, *T*. *simiae*, *T*. *vivax* and *T*. *congolense* and *T*. *godfreyi* ITS1 copies while CF/BR primers could detect 10^3^ *T*. *godfreyi* and *T*. *vivax* ITS1 copies, 10^4^ *T*. *simiae* and *T*. *congolense* ITS1 copies and 10^5^ *T*. *brucei* ITS1 copies. Trypanosomes have about 115 copies of ribosomal RNA genes [21].

### Read data and replicate analysis

**Fig 3:**
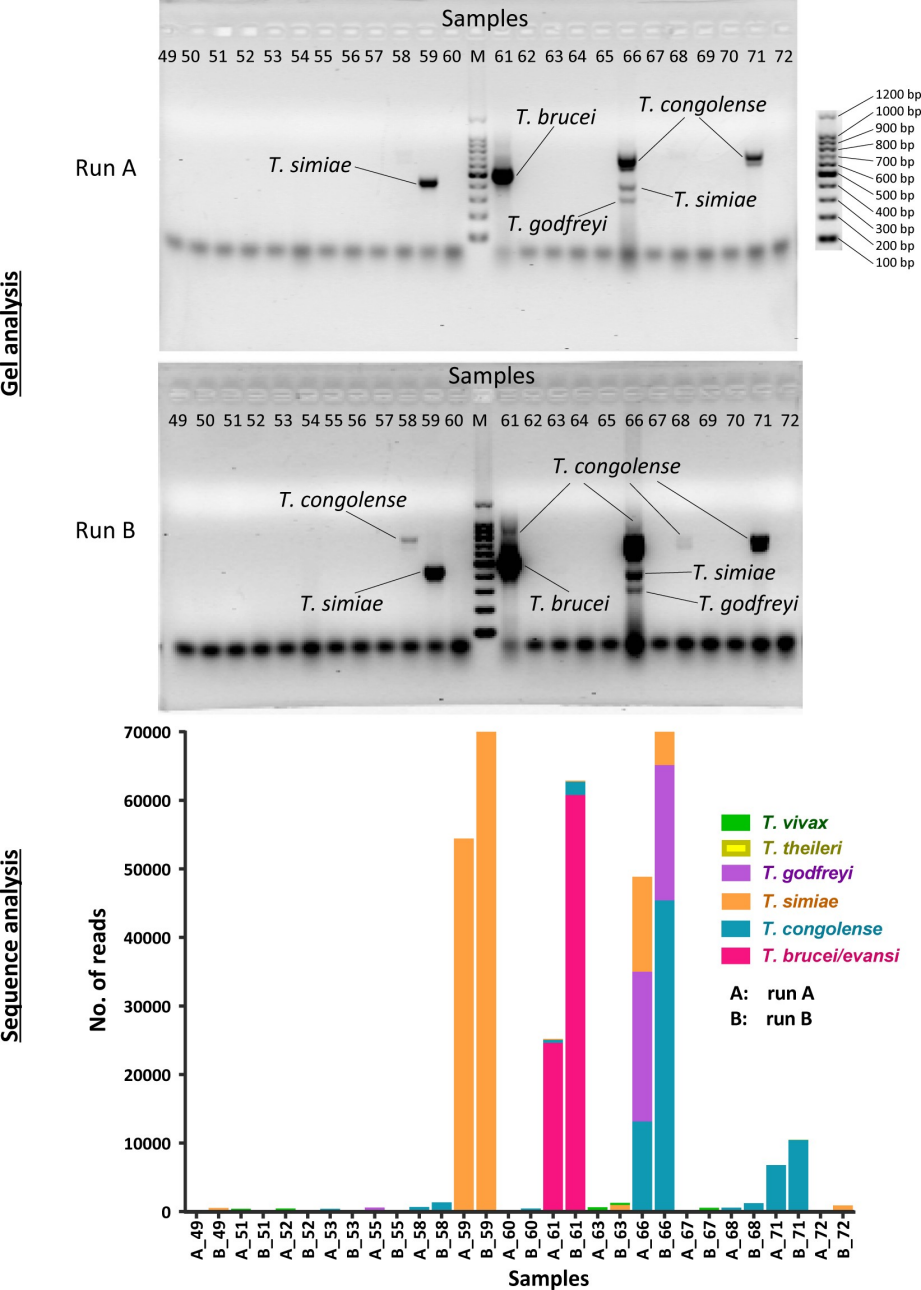
Representative replicates analysis results. (A) Gel analysis of Rufunsa samples done in replicate showing matching bands per sample. (B) Amplicon sequence analysis of the same samples in showing number of reads detected per species in each sample.

Reads generated from amplicon sequencing were of relatively good quality. Apart from those from Zimbabwe, more than 90% of the reads passed quality filtering in all samples (Table 2). The no. of ASVs generated in replicate runs was slightly different indicating slightly different detection sensitivities in the replicate PCR runs. Only the forward read was retained for downstream analysis in reads that did not merge due to either amplicon being longer than 600 b.p or due to low-quality bases in the overlap bases. This did not affect the final identification of reads as shown by the simulated data results described later. We analyzed the Rufunsa samples in replicates and compared the results. Both replicates had similar results in regard to individual Trypanosome species detection per sample seen in the gel image analysis (Fig 3A) as well as amplicon read analysis (Fig 3B). The outcome of detection for each of the Trypanosome species and subspecies in replicate runs was comparable and the Fischer’s exact test confirmed that there was no significant difference (*P<0*.*05*) in the number of positive detections in replicate runs (S2 Table).

**Table 2:** Read data of all samples analyzed.

Source of sample	No. of samples	Total no. of reads	Reads after pre-processing (% of total)	Raw ASVs	OTUs (97% clustering of ASVs)	ASVs post-filtering
Rufunsa Run A	200	916,055	897,598 (99.8%)	269	89	174
Rufunsa Run B	200	1,289,667	1,248,934 (94.8%)	320	95	232
Kafue	85	483,589	454,799 (91.4%)	131	48	56
Hurungwe	188	29,798	11,247 (79.5%)	137	63	116

Amplicon sequence variants (ASVs) generated were filtered to remove underrepresented and/or artifact ASVs from the final taxonomy table.

### Pipeline validation and accuracy of detection

Simulation of data generated from Trypanosome sequences downloaded from NCBI and analyzed using the AMPtk (amplicon toolkit) pipeline (version 1.2.4) (https://github.com/nextgenusfs/amptk) showed that amplicon sequence variants (ASVs) generated by the pipeline as primary units of representing sequence diversity, were more accurate in correctly inferring the diversity sequences compared to operational taxonomic units (OTUs) derived from clustering sequences at 97% identity (S3 Table). The specificity and precision of distinguishing between individual sequences of the same Trypanosome species are reflected by the number of ASVs or OTUs representing each of the different species. For example, only one OTU was generated for all three *Trypanosoma theileri* sequences, and three OTUs were generated for seven *Trypanosoma simiae* sequences, while the number of ASVs generated in each case represented each sequence accurately. The simulated data results indicated that read analysis using the AMPtk pipeline and ASVs instead of OTUs was suitable for sensitive identification of Trypanosome reads.

### Amplicon sequencing improves the sensitivity of detection and reveals errors of detection in conventional ITS1 PCR-gel analysis

**Fig 4:**
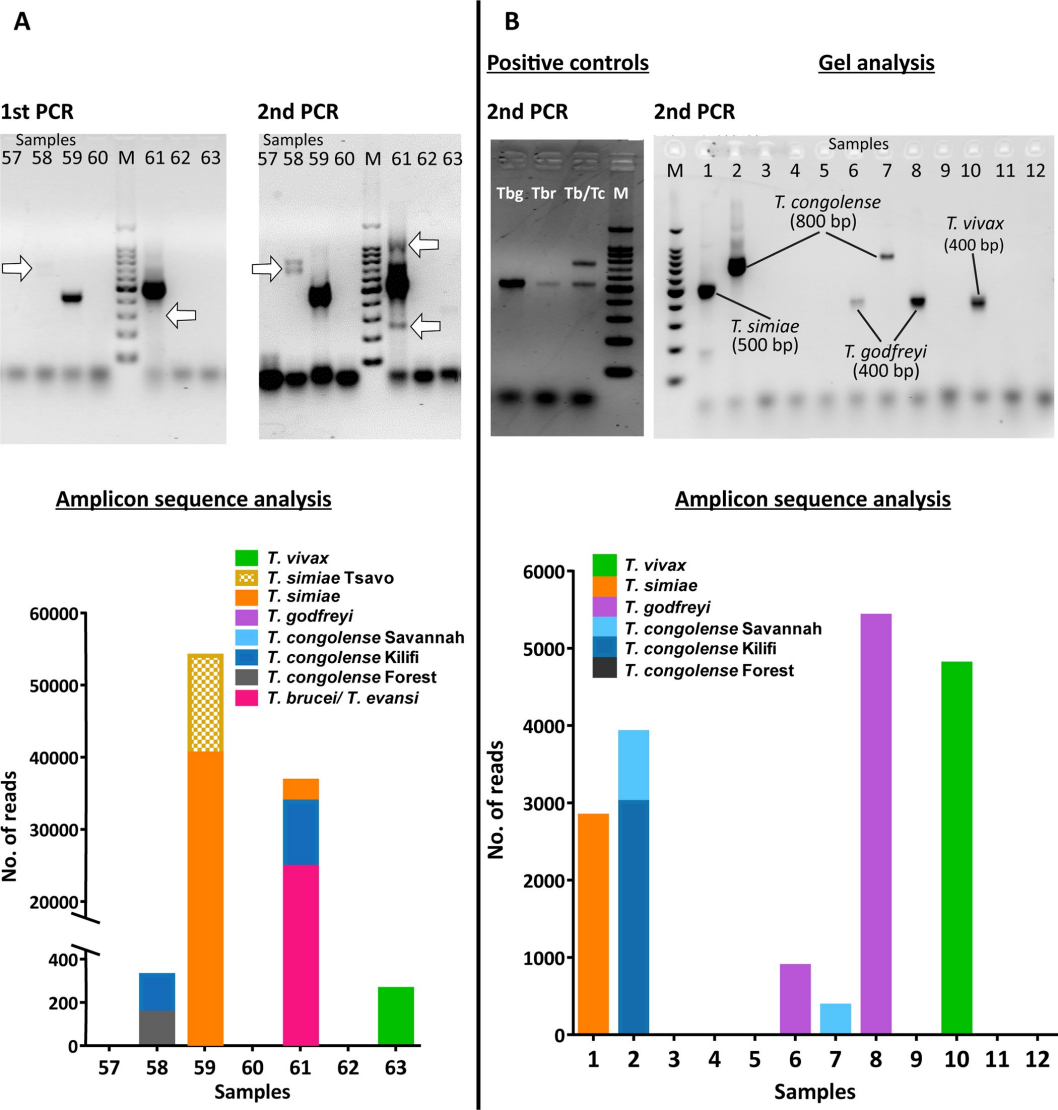
Representative gel and sequence analysis results. (A) Arrows showing bands are not visible after the 1st PCR become visible after 2nd PCR. (B) By gel analysis, amplicon bands of samples 5, 7 and 10 are indistinguishable by size and are deemed to be all *T*. *godfreyi* while sequencing reveals that the amplicon of sample 10 is, in fact, *T*. *vivax*. Positive controls comprise; Tbg (*T*. *brucei gambiense*), Tbr (*T*. *brucei rhodesiense*), Tb/Tc (an artificial mixture of equal amounts of *T*. *brucei gambiense* and *T*. *congolense* DNA).

By comparing gel images after PCR and sequence data, it was observed that the sensitivity of detection of Trypanosome DNA was increased by sequencing. Samples with bands that were barely visible after the 1^st^ PCR became visible after the 2^nd^ PCR and were confirmed as positive after sequencing (Fig 4A). It was also observed that some *T*. *godfreyi* and *T*. *vivax* amplicon bands were of a relatively similar size and it was difficult to distinguish the two by gel analysis alone (Fig 4B). From this example, sample no. 10 has an ITS amplicon size of about 400 b.p similar to that of sample no. 6 and 8. Sequence analysis showed that the band in sample no. 10 was identified as *T*. *vivax* while bands observed in sample no. 6 and 8 were identified as *T*. *godfreyi* despite their similar sizes. Mixed and single infections with multiple and single bands respectively were observed and confirmed by amplicon sequence analysis. Results for the second PCR using dual-index primers showed consistency with those of the first PCR. There were no bands visible outside the expected range indicating the absence of non-specific amplification in both PCR steps. The 1^st^ PCR amplicons were slightly longer than expected sizes due to the adapter sequences (approx. 80 bp) added to the primer, therefore the bands observed corresponded to *T*. *congolense (Kilifi/Forest and Savannah);* 650–800 b.p, *T*. *brucei;* 520–540 bp, *T*. *simiae;* 440–500 bp, *T*. *godfreyi;* 320–400 bp, and *T*. *vivax;* 290–400 bp.

### Trypanosome ITS1 sequences can be used to distinguish between different Trypanosome species and subspecies but not for the Trypanozoon subgenus

**Fig 5:**
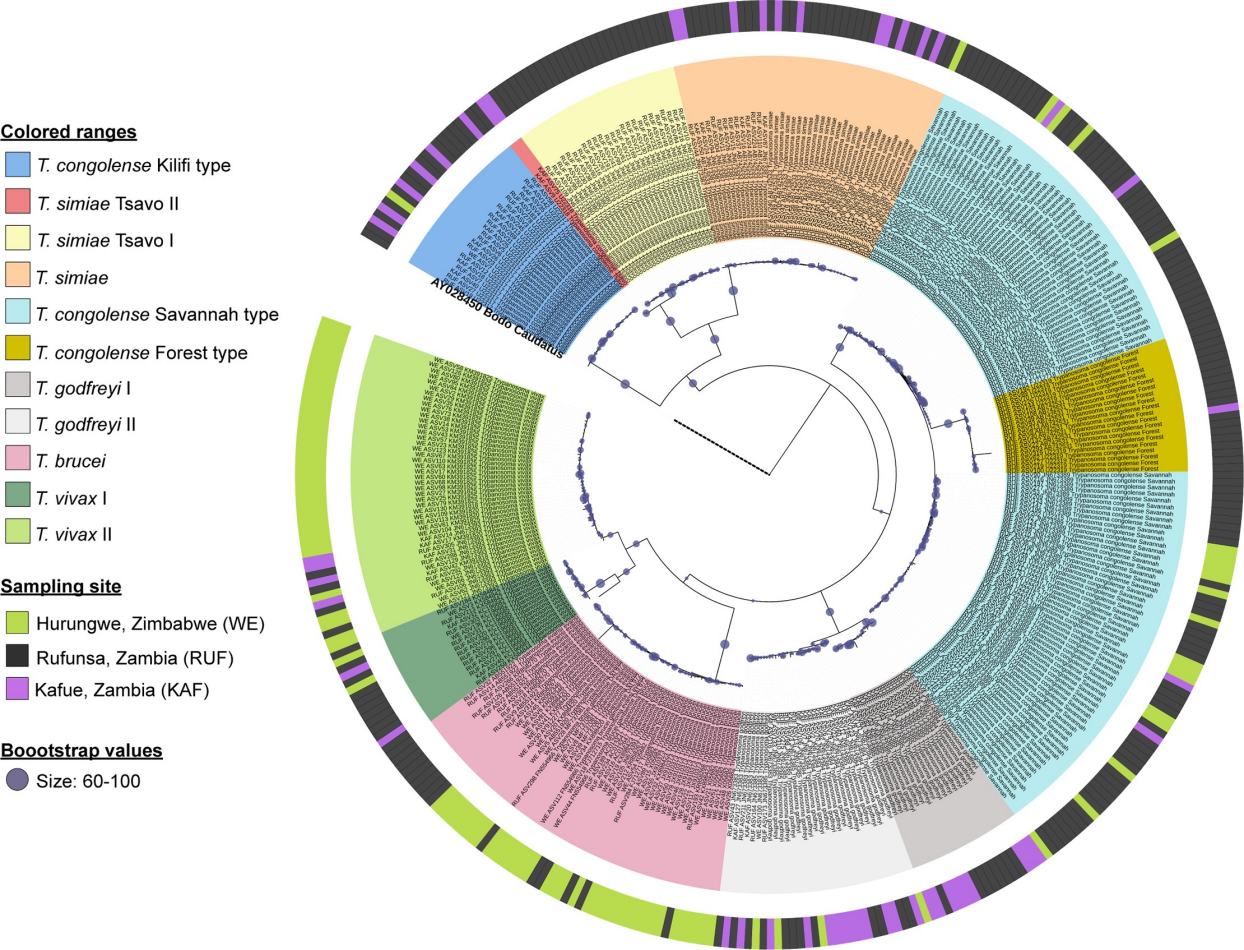
Phylogenetic tree of unique ASVs generated from amplicon sequence data. A *Bodo caudatus* ITS1 sequence was included as outgroup. Individual Trypanosome species and subspecies cluster into distinct clades. ASV are named after their respective blast best hit matches.

The accuracy in distinguishing between Trypanosome species and subspecies was analyzed by phylogenetic analysis of ASV sequences and their species identity allocated by BLAST. ASVs were named after the area of collection of the sample they originated from, ASV number allocated during analysis, accession number and the taxonomic name of their respective top hit BLAST subject sequence. Phylogenetic analysis of all ASVs obtained from this study showed that ASVs named after same Trypanosome species clustered together regardless of sample collection location. Sub-clustering into different subspecies of the same species was also observed (Fig 5). The *Nannomonas* subgenus showed the highest diversity of sub-clustering where *T*. *simiae* clustered into two main subspecies; *T*. *simiae* and *T*. *simiae Tsavo*. Two *T*. *simiae* Tsavo II ASVs from Kafue, with 91% and 97% identity to *T*. *congolense* Tsavo (Accession number U22318) recently reviewed and classified as *T*. *simiae* Tsavo [49,50] clustered distinctly from the rest of the *T*. *simiae* Tsavo I ASVs. *T*. *congolense* ASVs showed the highest diversity and clustered into three main subspecies; Kilifi, Riverine/Forest, and Savannah. *T*. *congolense* Savannah represented the most diversity in all the ASVs analyzed from all the samples. *T*. *congolense* Kilifi clustered separately and far from *T*. *congolense* Savannah and Riverine/Forest subspecies. *T*. *godfreyi* showed sub-clustering into two main subspecies while *T*. *vivax* (belonging to the *Dutonella* subgenus) also clustered into two subspecies. It was expected that the *Trypanozoon* subgenus (*T*. *brucei/T*. *evansi*) did not show any distinct sub-clustering.

### Prevalence and distribution of Trypanosome species in tsetse flies

**Fig 6:**
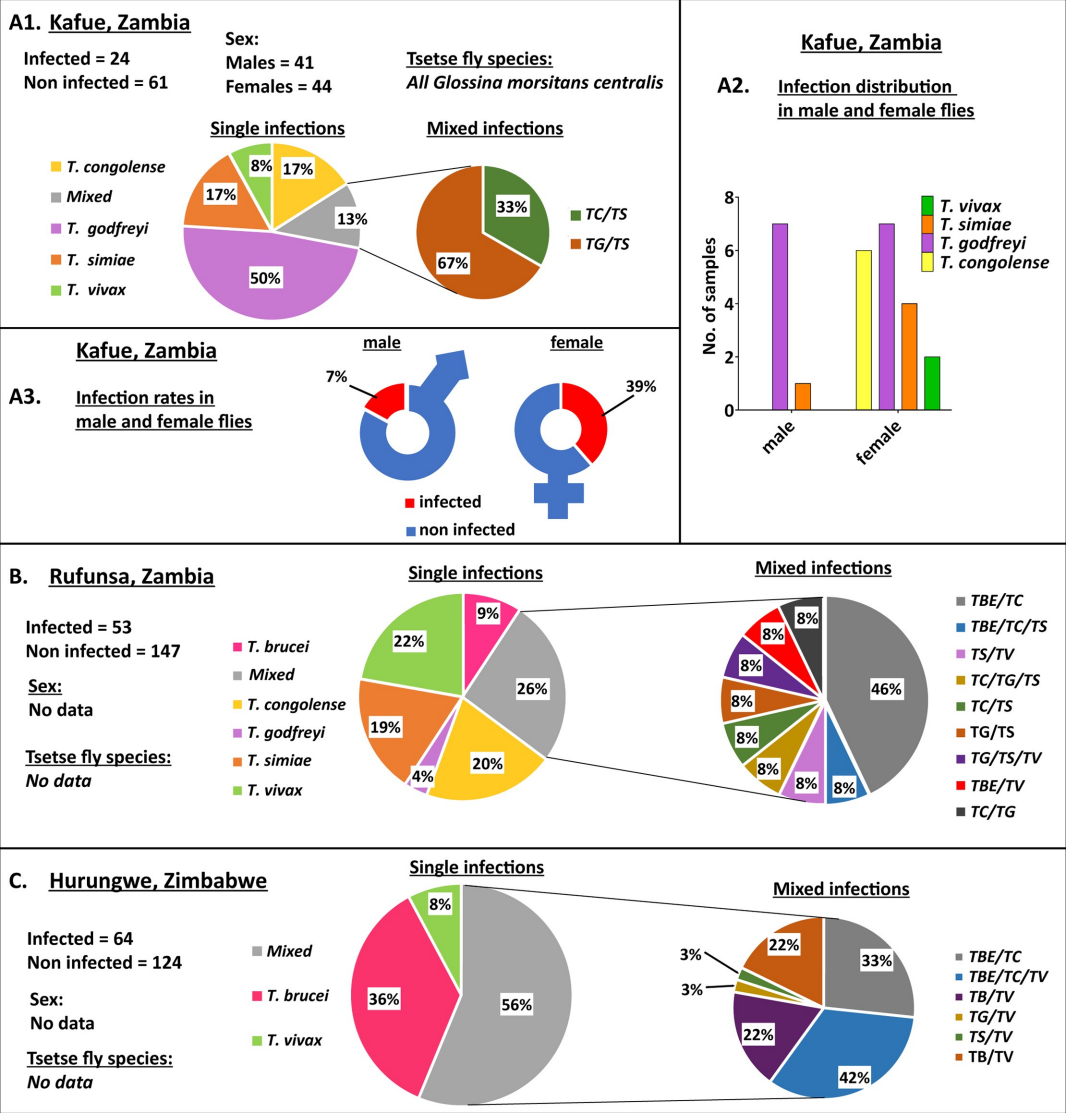
The distribution of Trypanosome species amongst infected tsetse flies. (A1) Pie chart showing the prevalence of Trypanosome species in wild-caught tsetse flies collected from Kafue, Zambia. (A2) Bar graph showing the Trypanosome species infecting male and female flies from flies collected in Kafue, Zambia. (A3) Graphical representation showing the infection rates of male and female flies collected in Kafue, Zambia. (B) Pie chart showing the prevalence of Trypanosome species in wild-caught tsetse flies collected from Hurungwe, Zimbabwe. (C) Pie chart showing the prevalence of Trypanosome species in wild-caught tsetse flies collected from Rufunsa, Zambia. TBE = *T*. *brucei/T*. *evansi*, TV = *T*. *vivax*, TS = *T*. *simiae*, TG = *T*. *godfreyi*, and TC = *T*. *congolense*.

The prevalence of Trypanosome infection in tsetse flies caught in the Rufunsa area, Zambia, was 25.6%, that of in the Kafue area, also Zambia, 28.2%, while that of the Hurungwe area, Zimbabwe, was 47.3%. Flies caught in Rufunsa had the highest prevalence of *T*. *congolense* while those from Kafue had the highest prevalence of *T*. *godfreyi* (Table 3). The highest prevalence of *T*. *brucei/ T*. *evansi* was recorded in flies caught in Hurungwe. We did not detect any *T*. *brucei*/ *T*. *evansi* from flies collected in Kafue. Mixed infections were predominant in flies caught in Rufunsa and Hurungwe while flies caught in Kafue were predominantly infected with *T*. *godfreyi* (Fig 6). Only tsetse flies from the Kafue region were sorted by sex during collection and we observed that the infection rate in female flies (38.6%) was more than twice that of male flies (17.1%). Additionally, we did not detect *T*. *congolense* and *T*. *vivax* infections in male flies. Flies caught in Hurungwe did not have single infections with *T*. *congolense* or *T*. *godfreyi*.

**Table 3:** Prevalence of Trypanosome species infection in wild-caught tsetse flies.

Trypanosome species	Rufunsa (n = 200)	Kafue (n = 85)	Hurungwe (n = 188)
***Trypanozoon***	6.0%	0.00%	45.7%
	(3.5% - 10.2%)	0% - 4.3%)	(38.8%– 52.9%)
***T*. *congolense* Forest**	4.5%	1.2%	0.0%
	(2.4% - 8.3%)	(0.2% - 6.4%)	(0% - 2.0%)
***T*. *congolense* Kilifi**	7.5%	2.4%	4.8%
	(4.6% - 12.0%)	(0.7% - 8.2%)	(2.5% - 8.9%)
*T*. *congolense* Savannah	7.5%	4.7%	39.9%
	(4.6% - 12.0%)	(1.9% - 11.5%)	(33.2% - 47.0%)
***T*. *godfreyi***	3.0%	16.5%	3.7%
	(1.4% - 6.4%)	(10.1% - 25.8%)	(1.8% - 7.5%)
***T*. *simiae***	6.0%	5.9%	1.1%
	(3.5% - 10.2%)	(2.5% - 13.0%)	(0.3% - 3.8%)
***T*. *simiae* Tsavo**	8.7%	2.4%	0.0%
	(4.5% - 16.2%)	(0.7% - 8.2%)	(0% - 2.0%)
***T*. *vivax***	7.5%	2.4%	29.2%
	(4.6% - 12.0%)	(0.7% - 8.2%)	(23.2% - 36.1%)
**Trypanosoma**	26.5%	28.2%	47.3%
**(overall prevalence)**	(20.9% -33.0%)	(19.8% - 38.6%)	(40.3% - 54.5%)

Confidence levels at 95% for apparent prevalence (Wilson) are shown in brackets.

## Discussion

This study reports a new and versatile approach for detection of Trypanosome DNA in multiple samples with high sensitivity and precision than conventional PCR-gel approach. We have established that conventional ITS PCR gel analysis is not an accurate way of determining the prevalence of Trypanosome species infections since identification of species by band size is inaccurate and may lead to misidentification of some Trypanosome species. Our new approach is sensitive at the subspecies level and has a high capacity to process large amounts of samples in one run (approximately a 700 samples mixed library) owing to the high repertoire of Illumina dual indexing primers. However, we did not see any unique clusters that could distinguish between the *Trypanozoon* subspecies which are of high priority because 1) they cause HAT (*T*. *b rhodesiense* and *T*. *gambiense*) and 2) their distribution is not restricted to Africa (*T*. *evansi* and *T*. *equiperdum*). However, we did identify two clusters of *T*. *vivax*. This is important since *T*. *vivax* is distributed outside Africa since it can be transmitted both cyclically by tsetse flies and also mechanically. Failure to distinguish between *Trypanozoon* subspecies was expected since the ribosomal RNA genes are highly conserved in this subgenus and cannot be able to tell apart the subspecies [29,30]. Moreover, a study based on genome-wide SNP analysis of 56 *Trypanozoon* genomes, including eight *T*. *evansi* and four *T*. *equiperdum* has revealed extensively similar genomes [51]. A single molecular test able to distinguish between members of the *Trypanozoon* subspecies is yet to be developed thus, subspecies specific based tests remain obligatory for their identification. As part of this work, we have also developed new primers that show high sensitivity to *T*. *brucei* compared to conventional primers and cover a wider range of the *Trypanosoma* genus. With our approach, it is now possible to identify species and subspecies of Trypanosomes by sequence analysis on individual samples as opposed to pooled samples for a large dataset which allows for the detection of new isolates. It is also possible to make a better inference of the Trypanosome species circulating in an area. This approach is practical and, with the decreasing cost of next-generation sequencing, cost-effective way to monitor large field samples of all kinds. They can, therefore, be utilized in a wide range of samples from vectors and hosts and the analysis of new Trypanosome species.

The results obtained in this study indicate that *T*. *vivax* and *T*. *godfreyi* have very similarly sized ITS1 amplicons making it difficult to identify one from the other based solely on gel band sizes. Sequencing and clustering of the reads effectively address this issue.

Phylogenetic analysis shows several interesting population substructures in the cases of *T*. *simiae* and *T*. *congolense*. Within the *T*. *congolense* clade, Savannah and Riverine/Forest subspecies show more sequence similarity while the Kilifi type shows more divergence. This agrees with a previous study that found *T*. *congolense* Savannah and Riverine/Forest had 71% similarity in satellite DNA sequence [52] and that the Kilifi subspecies was as divergent from other *T*. *congolense* subspecies [53]. The clustering of *T*. *congolense* Kilifi close to *T*. *simiae* species than other *T*. *congolense* subspecies is quite interesting in that an earlier study had identified a new *T*. *congolense* Tsavo strain (Accession number U22318) [54] which has been classified as *T*. *simiae* Tsavo [55]. We identified two ASVs from Kafue area (classified as *T*. *simiae* Tsavo II in this study) that had 91% and 97% identity to the U22318 *T*. *congolense* Tsavo sequence and that clustered with *T*. *simiae* Tsavo rather than other *T*. *congolense* species sequences supporting the *T*. *simiae* Tsavo classification. However, they cluster separately from the other *T*. *simiae Tsavo* ASVs, suggesting that they may have a divergent genotype. Perhaps there is a complex relationship between *T*. *congolense* and *T*. *simiae* species yet to be identified.

Prevalence of Trypanosome infection in caught tsetse flies differed in the sampled areas with single and mixed infection being detected in flies caught agreeing with previous studies [37,56,57]. This may be an important factor in the exchange of information between species. We also observed that the infection rate of female tsetse flies was more than twice that of male flies. This result is in contrast to dissection data from the Tinde experiment where male *Glossina morsitans centralis* had a salivary gland infection rate (5.4%) more than twice that of females (2.1%) [58]. However, our results agree with other studies on *Glossina morsitans*, reporting high infection rates in female flies compared to males [59,60]. More research is needed to find out the role of sex and infection rate differences between the different *Glossina* species in both laboratory and wild caught flies.

To conclude, our results imply that with this approach, it is possible to detect and distinguish between different Trypanosome species and subspecies accurately (with the exception of *Trypanozoon* subgenus) and therefore infer prevalence of infection more precisely using a single test without having to undertake satellite DNA analysis that requires species-specific primers. This is made possible by deep sequencing which enables resolution at a single nucleotide level. This high resolution at sub-cluster level utilizing only the ITS1 region has not been shown before thus a practical and sensitive barcoding of African trypanosomes. Using our approach, it is thus possible to distinguish *T*. *godfreyi* from *T*. *vivax*, as well as highlight finer subpopulation structures within the *T*. *simiae* and *T*. *congolense* clades that raise interesting questions regarding their classification. It is highly likely that there are genomic and taxonomic differences between *T*. *vivax*, *T*. *godfreyi* and *T*. *congolense* subspecies that need to be studied. This could provide answers on the evolution of Trypanosomes such as; what contribution do these Trypanosome subspecies make to livestock disease? Are these genotypes responsible for assumed “strain” differences in drug response? Can these new genotypes be correlated with the old morphological criteria and species designations? Do these “strains” have the potential of evolving to new subspecies that could pose new risks? There is a need for more studies to catch up with the molecular taxonomy to answer these questions.

## Supporting information

S1 Fig The sensitivity of AITSF/AITR primers compared to CF/BR primers in the detection of Trypanosome ITS1 inserts cloned in the pGEMT-easy vector. (TIF)

S1 Text Script with all commands used to run the AMPtk pipeline. (PDF)

S1 Table Amplicon sizes of new primers (ATSF/AITSR) compared to other primers (CF/BR and ITS1/ITS2) obtained by simulated PCR. (PDF)

S2 Table Statistical analysis of detection of individual Trypanosome species in replicate runs. (PDF)

S3 Table Matrix comparison of ASVs and OTUs from simulated data. (PDF)
