# Supplementary figures and images for: A single test approach for accurate and sensitive detection and taxonomic characterization of Trypanosomes by comprehensive analysis of internal transcribed spacer 1 amplicons
Source: PLoS Negl Trop Dis. 2019 Feb 25;13(2):e0006842. doi: 10.1371/journal.pntd.0006842 (PMC6414030; doi:10.1371/journal.pntd.0006842)

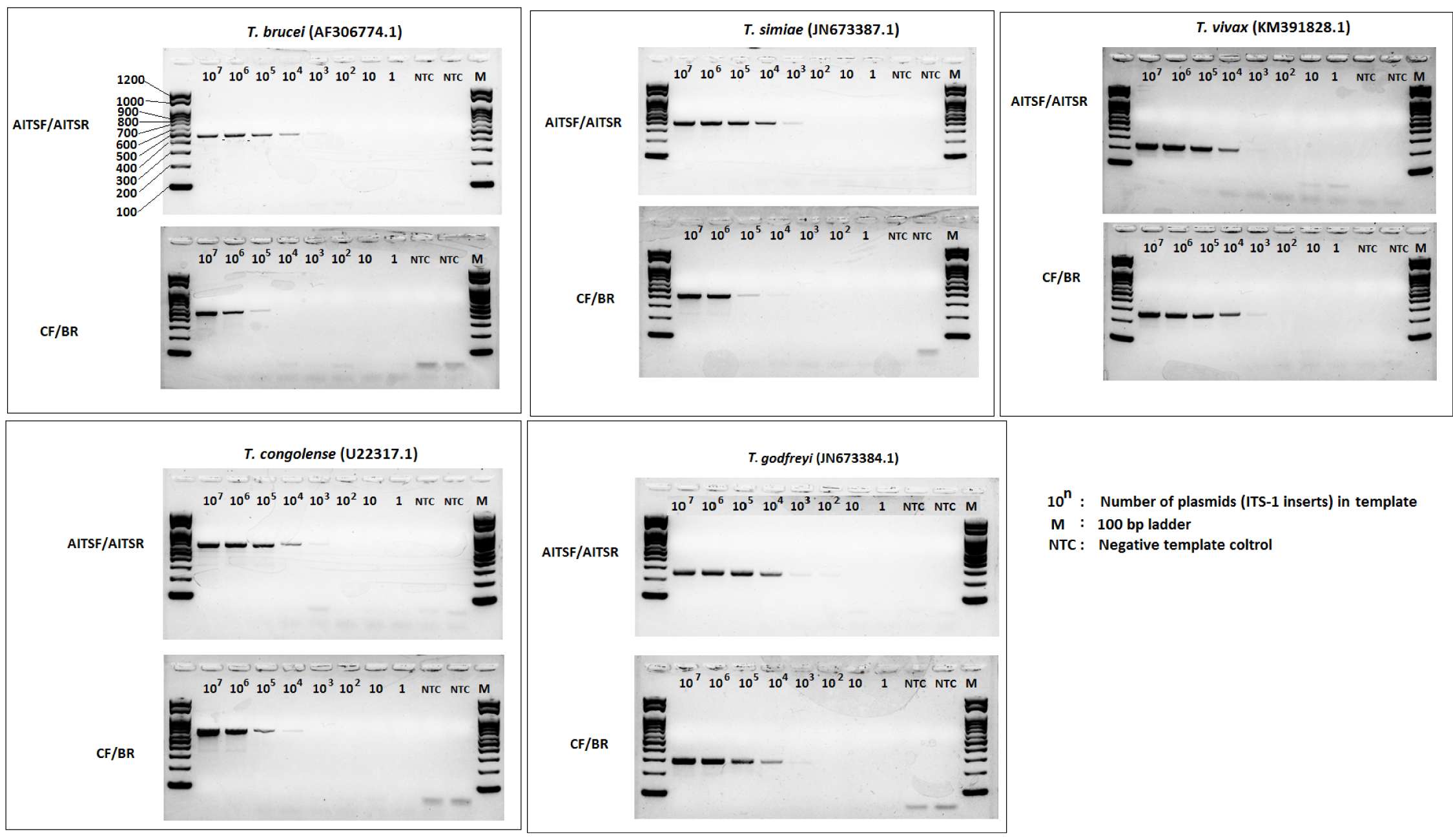

Supplement: S1 Fig — (TIF) [file pntd.0006842.s001.tif]
