# Supplementary material for: A single test approach for accurate and sensitive detection and taxonomic characterization of Trypanosomes by comprehensive analysis of internal transcribed spacer 1 amplicons
Source: PLoS Negl Trop Dis. 2019 Feb 25;13(2):e0006842. doi: 10.1371/journal.pntd.0006842 (PMC6414030; doi:10.1371/journal.pntd.0006842)
Supplement: S1 Text — (PDF) [file pntd.0006842.s002.pdf]

```
#!/usr/bin/bash

# source activate amptk
# Run using amptk v1.2.4
# usearch v10, vsearch v 2.8

DIR="<path/to/directory/containing/fastq_read_files>"
OUT="<OUT/of/analysis Output>"
mkdir $DIR/$OUT
cd $DIR/$OUT

# merge PE reads, length filter, remove PhiX reads, strip primers & create mapping file
# rescue foward reads if reads dont merge (default)

amptk illumina -i $DIR -o $OUT -f CGAAAGTTCACCGATATTGC -r AGGAAGCCAAGTCATCCATC --require_primer on -l 600 -u usearch10

# quality filter @ maxEE <8 (based on fastqc check), run DADA2 denoising, obtain ASVs & map reads to ASVs

amptk dada2 -i ./$OUT.demux.fq.gz -o $OUT --platform illumina -e 8 -u usearch10

# sorting ASVs, normalize ASV table & auto-detect index bleeding
# Default index-bleed percentage of 0.005

amptk filter -i ./$OUT.otu_table.txt -f ./$OUT.ASVs.fa -p 0.005 -u usearch10

# Extra filtering by Removing erroneous molecular ASVs
# Identifies errors by combining sequence similarity and co-occurrence patterns yielding reliable biodiversity estimates
# pairwise identity of ASVs @ 84%, filter ASVs by concurrence & abundance

amptk lulu -i ./$OUT.final.txt -o $OUT -f ./$OUT.filtered.otus.fa

# percentage identity > 85%, alignment coverage of hit to query > 95% & pick best hit
# blastn should be version 2.6.0; NCBI taxonomy file taxdb should be downloaded and added to PATH as BLASTDB

echo "Now Running Blastn Remotely"
blastn -db nt -query ./$OUT.lulu.otus.fa -out $OUT.blast.out -perc_identity 85 -qcov_hsp_perc 95 -num_alignments 1
-num_descriptions 1 -remote

grep RID $OUT.blast.out | uniq > $OUT.blast_rids.out

for i in $( cat  $OUT.blast_rids.out); do
        if [[ $i != "RID:" ]]; then
        echo "Now Running: $i"
        blast_formatter -rid $i -outfmt "6 qcovhsp pident score qseqid sacc scomOUTs stitle" -out $OUT.blastn$i.out
        fi
        done

cat $OUT.blastn* > $OUT.blastn_final.out

#Parse the blast result to make sure that query coverage alignment is > 60 and alignment score is >100

awk '(NR>1) && ($1 > 60) && ($3 > 100)' $OUT.blastn_final.out > $OUT.blast_parsed.txt

#Rename taxonomy of the species for known sub-types (information derived from publications) and repeated phylogenetic analysis.
awk -F "\t" '{OFS=FS}{ if ($5== "U22317") $6=$6" Kilifi"; print}' $OUT.blast_parsed.txt\
|awk -F "\t" '{OFS=FS}{ if ($5== "U22318") $6=$6" Tsavo"; print}'\
|awk -F "\t" '{OFS=FS}{ if ($5== "U22315") $6=$6" Savannah"; print}'\
|awk -F "\t" '{OFS=FS}{ if ($5== "JN673389") $6=$6" Savannah"; print}'\
|awk -F "\t" '{OFS=FS}{ if ($5== "JN673388") $6=$6" Savannah"; print}'\
|awk -F "\t" '{OFS=FS}{ if ($5== "FJ712718") $6=$6" Savannah"; print}'\
|awk -F "\t" '{OFS=FS}{ if ($5== "MG255203") $6=$6" Savannah"; print}'\
|awk -F "\t" '{OFS=FS}{ if ($5== "MG255204") $6=$6" Savannah"; print}'\
|awk -F "\t" '{OFS=FS}{ if ($5== "JX910374") $6=$6" Savannah"; print}'\
|awk -F "\t" '{OFS=FS}{ if ($5== "U22319") $6=$6" Forest"; print}'\
|awk -F "\t" '{OFS=FS}{ if ($5== "AB742531") $6=$6" Forest"; print}'\
|awk -F "\t" '{OFS=FS}{ if ($5== "JN673380") $6=$6" Tsavo"; print}'\
|awk -F "\t" '{OFS=FS}{ if ($5== "JN673381") $6=$6" Tsavo"; print}'\
|awk -F "\t" '{OFS=FS}{ if ($5== "JN673382") $6=$6" Tsavo"; print}'\
|awk -F "\t" '{OFS=FS}{ if ($5== "JN673379") $6=$6" Tsavo"; print}' > $OUT.blast_Subsp_Renamed.txt

#Select only trypanosoma species hits

awk '$6 ~ /Trypanosoma/' $OUT.blast_Subsp_Renamed.txt > $OUT.blast_Subsp_Renamed_trypsOnly.txt
cut -f 4,5,6 $OUT.blast_Subsp_Renamed_trypsOnly.txt > $OUT.blast_Out.final
sed 's/\t//g2' $OUT.blast_Out.final | sed 's/ //g' > $OUT.taxonomy.temp

#Remove 0 hit results
#Rename files to AMPtk agreeable taxonomy format

amptk taxonomy -f ./$OUT.lulu.otus.fa -i ./$OUT.lulu.otu_table.txt -o $OUT -m ./$OUT.mapping_file.txt -t $OUT.taxonomy.temp
--fasta_db ./$OUT.lulu.otus.fa  -u usearch10
grep -P '^(?!.*Hit).*$' $OUT.otu_table.taxonomy.txt > $OUT.otu_tax_table.temp
awk  -vOFS='\t' '{$(NF)=""; print $0}' $OUT.otu_tax_table.temp | sed 's/\tID/ ID/g'| sed 's/\t$//' > $OUT.otu_tax_table.new
sed 's/\t/|/g2' $OUT.blast_Out.final | sed 's/|Trypanosoma /;g:Trypanosoma /g'> $OUT.taxonomy.txt

#Run taxonomy command to create taxonomy labelled OTU table

amptk taxonomy -f ./$OUT.lulu.otus.fa -i $OUT.otu_tax_table.new -o $OUT -m ./$OUT.mapping_file.txt -o $OUT -t
./$OUT.taxonomy.txt --fasta_db ./$OUT.lulu.otus.fa -u usearch10

#Generate ASV fasta file per species

awk '/^>/ { p = ($0 ~ /Trypanosoma/)} p' $OUT.otus.taxonomy.fa > $OUT.otus.taxonomy2.fa
awk '/^>/ { p = ($0 ~ /brucei/)} p' $OUT.otus.taxonomy.fa > $OUT.otus.brucei.fa
awk '/^>/ { p = ($0 ~ /congolense/)} p' $OUT.otus.taxonomy.fa > $OUT.otus.congolense.fa
awk '/^>/ { p = ($0 ~ /simiae/)} p' $OUT.otus.taxonomy.fa > $OUT.otus.simiae.fa
awk '/^>/ { p = ($0 ~ /godfreyi/)} p' $OUT.otus.taxonomy.fa > $OUT.otus.godfreyi.fa
awk '/^>/ { p = ($0 ~ /vivax/)} p' $OUT.otus.taxonomy.fa > $OUT.otus.vivax.fa
```
