# Supplementary material for: A single test approach for accurate and sensitive detection and taxonomic characterization of Trypanosomes by comprehensive analysis of internal transcribed spacer 1 amplicons
Source: PLoS Negl Trop Dis. 2019 Feb 25;13(2):e0006842. doi: 10.1371/journal.pntd.0006842 (PMC6414030; doi:10.1371/journal.pntd.0006842)
Supplement: S1 Table — (PDF) [file pntd.0006842.s003.pdf]

**Trypanosome species/sub-species**	**AITSF/R Amplicon lengths (b.p)**	**CF/BR Amplicon lengths (b.p)**	**ITS1/ITS2 Amplicon lengths (b.p)**
Trypanaosoma congolense other isolates	633 - 705	686 - 717	600 - 674
Trypanaosoma congolense riverine/forest-type	660	713	-
Trypanaosoma congolense Kilifi-type	560	613	529
Trypanosoma cruzi	544 - 570	-	512 - 518
Trypanosoma rangeli	525	-	455 - 494
Trypanosoma cf. varani	511	-	480
Trypanosoma kuseli	504 - 506	-	473
Trypanosoma otospermophili	504	-	472
Trypanosoma lewisi	504	-	473 - 475
Trypanaosoma grosi	424 - 435	-	401 - 404
Trypanaosoma brucei/ evansi	415 - 431	468 - 484	384 - 394
Trypanosoma simiae	331 - 343	369-397	300 - 319
Trypanosoma cf. cervi	333 - 340	-	303 - 312
Trypanosoma avium	322	-	291
Trypanaosoma congolense Tsavo-type	316	369	285
Trypanaosoma theileri	269 - 350	-	238 - 265
Trypanosoma godfreyi	220	273	189
Trypanaosoma vivax	226-238	248 - 264	164 - 187
