# Supplementary material for: A single test approach for accurate and sensitive detection and taxonomic characterization of Trypanosomes by comprehensive analysis of internal transcribed spacer 1 amplicons
Source: PLoS Negl Trop Dis. 2019 Feb 25;13(2):e0006842. doi: 10.1371/journal.pntd.0006842 (PMC6414030; doi:10.1371/journal.pntd.0006842)
Supplement: S2 Table — (PDF) [file pntd.0006842.s004.pdf]

Species/Sub-group	Run	Positive	Negative	Fisher's exact test P value Two-sided	P value summary (alpha<0.05)
T. brucei/T. evansi	RunA	8	192	0.4923	ns
	RunB	12	188		
T. congolense Forest	RunA	7	193	0.7996	ns
	RunB	9	191		
T. congolense Kilifi	RunA	12	188	0.6909	ns
	RunB	15	185		
T. congolense Savannah	RunA	14	186	1	ns
	RunB	15	185		
T. godfreyi	RunA	14	186	0.1062	ns
	RunB	6	194		
T. simiae	RunA	9	191	0.6549	ns
	RunB	12	188		
T. simiae Tsavo	RunA	9	191	1	ns
	RunB	8	192		
T. vivax	RunA	10	190	0.4092	ns
	RunB	15	185		
