# Supplementary material for: A single test approach for accurate and sensitive detection and taxonomic characterization of Trypanosomes by comprehensive analysis of internal transcribed spacer 1 amplicons
Source: PLoS Negl Trop Dis. 2019 Feb 25;13(2):e0006842. doi: 10.1371/journal.pntd.0006842 (PMC6414030; doi:10.1371/journal.pntd.0006842)
Supplement: S3 Table — (PDF) [file pntd.0006842.s005.pdf]

OTU Taxonomy	AB175624 Trypanosoma grosi	AB175626 Trypanosoma kuseli	AB362411 Trypanosoma minasense	Y00055 Crithidia fasciculata	AY028450 Parabodo caudatus	JN673396 Trypanosoma theileri			JN673391 Trypanosoma brucei		JN673390 Trypanosoma brucei	U22320 Trypanosoma simiae	JN673386 Trypanosoma simiae	JN673387 Trypanosoma simiae	JN673382 Trypanosoma simiae Tsavo				JN673384 Trypanosoma godfreyi	JN673385 Trypanosoma godfreyi	MG255220 Trypanosoma vivax	JN673392 Trypanosoma vivax	JN673393 Trypanosoma vivax	JN673394 Trypanosoma vivax	U22317 Trypanosoma congolense Kilifi	U22319 Trypanosoma congolense Forest	JN673388 Trypanosoma congolense Savannah	JN673389 Trypanosoma congolense Savannah	JN673398 Trypanosoma sp. Z3206	JN673399 Trypanosomatidae sp. TS07016	JN673400 Trypanosomatidae sp. Z26907	JN673401 Trypanosomatidae sp. Z1505	JN673402 Trypanosomatidae sp. TS06050	JN673403 Trypanosomatidae sp. TS07116
OTU ID	OTU19	OTU1	OTU12	OTU20	OTU6	OTU28			OTU17		OTU18	OTU25	OTU22	OTU23	OTU24				OTU3	OTU2	OTU5	OTU9	OTU11	OTU8	OTU13	OTU15	OTU10	OTU4	OTU26	OTU23	OTU27	OTU7	OTU16	OTU14
ASV Taxonomy	AB175624 Trypanosoma grosi	AB175626 Trypanosoma kuseli	AB362411 Trypanosoma minasense	Y00055 Crithidia fasciculata	AY028450 Parabodo caudatus	JN673395 Trypanosoma theileri	JN673396 Trypanosoma theileri	JN673397 Trypanosoma theileri	AF306771 Trypanosoma brucei	JN673391 Trypanosoma brucei	JN673390 Trypanosoma brucei	U22320 Trypanosoma simiae	JN673386 Trypanosoma simiae	JN673387 Trypanosoma simiae	JN673379 Trypanosoma simiae Tsavo	JN673380 Trypanosoma simiae Tsavo	JN673381 Trypanosoma simiae Tsavo	JN673382 Trypanosoma simiae Tsavo	JN673384 Trypanosoma godfreyi	JN673385 Trypanosoma godfreyi	MG255220 Trypanosoma vivax	JN673392 Trypanosoma vivax	JN673393 Trypanosoma vivax	JN673394 Trypanosoma vivax	U22317 Trypanosoma congolense Kilifi	U22319 Trypanosoma congolense Forest	JN673388 Trypanosoma congolense Savannah	JN673389 Trypanosoma congolense Savannah	JN673398 Trypanosoma sp. Z3206	JN673399 Trypanosomatidae sp. TS07016	JN673400 Trypanosomatidae sp. Z26907	JN673401 Trypanosomatidae sp. Z1505	JN673402 Trypanosomatidae sp. TS06050	JN673403 Trypanosomatidae sp. TS07116
Representative Sequences / ASV ID	ASV19	ASV1	ASV12	ASV21	ASV6	ASV34	ASV33	ASV32	ASV20	ASV17	ASV18	ASV26	ASV23	ASV24	ASV28	ASV27	ASV25	ASV29	ASV3	ASV2	ASV5	ASV9	ASV11	ASV8	ASV13	ASV15	ASV10	ASV4	ASV30	ASV22	ASV31	ASV7	ASV16	ASV14
AB175624TrypanosomaGrosi	10000	0	0	0	0	0	0	0	0	0	0	0	0	0	0	0	0	0	0	0	0	0	0	0	0	0	0	0	0	0	0	0	0	0
AB175626TrypanosomaKuseli	0	9992	0	0	0	0	0	0	0	0	0	0	0	0	0	0	0	0	0	0	0	0	0	0	0	0	0	0	0	0	0	0	0	0
AB362411TrypanosomaMinasense	0	0	9970	0	0	0	0	0	0	0	0	0	0	0	0	0	0	0	0	0	0	0	0	0	0	0	0	0	0	0	0	0	0	0
Y00055CrithidiaFasciculata	0	0	0	9999	0	0	0	0	0	0	0	0	0	0	0	0	0	0	0	0	0	0	0	0	0	0	0	0	0	0	0	0	0	0
AY028450BodoCaudatus	0	0	0	0	9793	0	0	0	0	0	0	0	0	0	0	0	0	0	0	0	0	0	0	0	0	0	0	0	0	0	0	0	0	0
JN673395TrypanosomaTheileriPuku	0	0	0	0	0	9928	0	0	0	0	0	0	0	0	0	0	0	0	0	0	0	0	0	0	0	0	0	0	0	0	0	0	0	0
JN673396TrypanosomaTheileriPuku	0	0	0	0	0	0	9998	0	0	0	0	0	0	0	0	0	0	0	0	0	0	0	0	0	0	0	0	0	0	0	0	0	0	0
JN673397TrypanosomaTheileriPuku	0	0	0	0	0	0		9949	0	0	0	0	0	0	0	0	0	0	0	0	0	0	0	0	0	0	0	0	0	0	0	0	0	0
AF306771TrypanosomaBrucei	0	0	0	0	0	0	0	0	9997	0	0	0	0	0	0	0	0	0	0	0	0	0	0	0	0	0	0	0	0	0	0	0	0	0
JN673391TrypanosomaBruceiSpottedhyena	0	0	0	0	0	0	0	0	0	10000	0	0	0	0	0	0	0	0	0	0	0	0	0	0	0	0	0	0	0	0	0	0	0	0
JN673390TrypanosomaBruceiZebra	0	0	0	0	0	0	0	0	0	0	9997	0	0	0	0	0	0	0	0	0	0	0	0	0	0	0	0	0	0	0	0	0	0	0
U22320TrypanosomaSimiae	0	0	0	0	0	0	0	0	0	0	0	9997	0	0	0	0	0	0	0	0	0	0	0	0	0	0	0	0	0	0	0	0	0	0
JN673386TrypanosomaSimiaeWarthog	0	0	0	0	0	0	0	0	0	0	0	0	9993	0	0	0	0	0	0	0	0	0	0	0	0	0	0	0	0	0	0	0	0	0
JN673387TrypanosomaSimiaeWarthog	0	0	0	0	0	0	0	0	0	0	0	0	0	9996	0	0	0	0	0	0	0	0	0	0	0	0	0	0	0	0	0	0	0	0
JN673379TrypanosomaSimiaeTsavoWarthog	0	0	0	0	0	0	0	0	0	0	0	0	0	0	9997	0	0	0	0	0	0	0	0	0	0	0	0	0	0	0	0	0	0	0
JN673380TrypanosomaSimiaeTsavoWarthog	0	0	0	0	0	0	0	0	0	0	0	0	0	0	0	9995	0	0	0	0	0	0	0	0	0	0	0	0	0	0	0	0	0	0
JN673381TrypanosomaSimiaeTsavoWarthog	0	0	0	0	0	0	0	0	0	0	0	0	0	0	0	0	9997	0	0	0	0	0	0	0	0	0	0	0	0	0	0	0	0	0
JN673382TrypanosomaSimiaeTsavoWarthog	0	0	0	0	0	0	0	0	0	0	0	0	0	0	0	0	0	9999	0	0	0	0	0	0	0	0	0	0	0	0	0	0	0	0
JN673384TrypanosomaGodfreyiWarthog	0	0	0	0	0	0	0	0	0	0	0	0	0	0	0	0	0	0	9942	0	0	0	0	0	0	0	0	0	0	0	0	0	0	0
JN673385TrypanosomaGodfreyiWarthog	0	0	0	0	0	0	0	0	0	0	0	0	0	0	0	0	0	0	0	9954	0	0	0	0	0	0	0	0	0	0	0	0	0	0
U22316TrypanosomaVivax	0	0	0	0	0	0	0	0	0	0	0	0	0	0	0	0	0	0	0	0	9787	0	0	0	0	0	0	0	0	0	0	0	0	0
JN673392TrypanosomaVivaxGiraffe	0	0	0	0	0	0	0	0	0	0	0	0	0	0	0	0	0	0	0	0	0	9809	0	0	0	0	0	0	0	0	0	0	0	0
JN673393TrypanosomaVivaxWaterbuck	0	0	0	0	0	0	0	0	0	0	0	0	0	0	0	0	0	0	0	0	0	0	9769	0	0	0	0	0	0	0	0	0	0	0
JN673394TrypanosomaVivaxCapeBuffalo	0	0	0	0	0	0	0	0	0	0	0	0	0	0	0	0	0	0	0	0	0	0	0	9782	0	0	0	0	0	0	0	0	0	0
U22317TrypanosomaCongolenseKilifi	0	0	0	0	0	0	0	0	0	0	0	0	0	0	0	0	0	0	0	0	0	0	0	0	9992	0	0	0	0	0	0	0	0	0
U22319TrypanosomaCongolenseForest	0	0	0	0	0	0	0	0	0	0	0	0	0	0	0	0	0	0	0	0	0	0	0	0	0	9990	0	0	0	0	0	0	0	0
JN673388TrypanosomaCongolenseSpottedhyena	0	0	0	0	0	0	0	0	0	0	0	0	0	0	0	0	0	0	0	0	0	0	0	0	0	0	9994	0	0	0	0	0	0	0
JN673389TrypanosomaCongolenseSavannahLion	0	0	0	0	0	0	0	0	0	0	0	0	0	0	0	0	0	0	0	0	0	0	0	0	0	0	0	9990	0	0	0	0	0	0
JN673398TrypanosomaSPHippopotamus	0	0	0	0	0	0	0	0	0	0	0	0	0	0	0	0	0	0	0	0	0	0	0	0	0	0	0	0	9994	0	0	0	0	0
JN673399TrypanosomatidaeSPSpottedhyena	0	0	0	0	0	0	0	0	0	0	0	0	0	0	0	0	0	0	0	0	0	0	0	0	0	0	0	0	0	10000	0	0	0	0
JN673400TrypanosomatidaeSPCapeBuffalo	0	0	0	0	0	0	0	0	0	0	0	0	0	0	0	0	0	0	0	0	0	0	0	0	0	0	0	0	0	0	9996	0	0	0
JN673401TrypanosomatidaeSPHippopotamus	0	0	0	0	0	0	0	0	0	0	0	0	0	0	0	0	0	0	0	0	0	0	0	0	0	0	0	0	0	0	0	9992	0	0
JN673402TrypanosomatidaeSPWildebeest	0	0	0	0	0	0	0	0	0	0	0	0	0	0	0	0	0	0	0	0	0	0	0	0	0	0	0	0	0	0	0	0	9933	0
JN673403TrypanosomatidaeSPWildebeest	0	0	0	0	0	0	0	0	0	0	0	0	0	0	0	0	0	0	0	0	0	0	0	0	0	0	0	0	0	0	0	0	0	9956
